# Role of clinical, functional and social factors in the association between multimorbidity and quality of life: Findings from the Survey of Health, Ageing and Retirement in Europe (SHARE)

**DOI:** 10.1371/journal.pone.0240024

**Published:** 2020-10-20

**Authors:** Tatjana T. Makovski, Gwenaëlle Le Coroller, Polina Putrik, Yun Hee Choi, Maurice P. Zeegers, Saverio Stranges, Maria Ruiz Castell, Laetitia Huiart, Marjan van den Akker

**Affiliations:** 1 Department of Population Health, Luxembourg Institute of Health, Strassen, Luxembourg; 2 Department of Family Medicine, Care and Public Health Research Institute, Maastricht University, Maastricht, The Netherlands; 3 Chairgroup of Complex Genetics and Epidemiology, Nutrition and Metabolism in Translational Research, Care and Public Health Research Institute, Maastricht University, Maastricht, The Netherlands; 4 Competence Centre for Methodology and Statistics, Department of Population Health, Luxembourg Institute of Health, Strassen, Luxembourg; 5 Department of Health Services Research, Care and Public Health Research Institute, Maastricht University, Maastricht, The Netherlands; 6 Department of Epidemiology & Biostatistics, Western University, London, Ontario, Canada; 7 Department of Family Medicine, Western University, London, Ontario, Canada; 8 Academic Centre for General Practice / Department of Public Health and Primary Care, Katholieke Universiteit Leuven, Leuven, Belgium; 9 Institute of General Practice, Johann Wolfgang Goethe University, Frankfurt am Main, Germany; Sciensano, BELGIUM

## Abstract

**Objective:**

An increasing number of diseases is linked to deterioration of quality of life (QoL). Part of this association can be explained by socio-economic factors, which are most commonly accounted for. Our aim was to explore the potential contribution of other factors related to clinical burden, social interaction and functioning.

**Methods:**

A cross-sectional analysis was conducted on wave 6 of the population-based Survey of Health, Ageing and Retirement in Europe (SHARE), among participants aged 50+ (n = 67 179). The Control, Autonomy, Self-Realization and Pleasure (CASP-12v1) questionnaire measured QoL. The association between number of diseases and QoL was tested in a mixed-effects linear regression model. The base model controlled for socio-economic characteristics. Factors of interest (symptoms, polypharmacy, unmet care needs, utilisation of care, social network, personal and financial help, loneliness and activities of daily living (ADL) with instrumental activities (IADL)) were added to the base model one at a time and tested for relevance (i.e. change in the β-coefficient of the number of conditions of 15% or more).

**Results:**

Symptoms, polypharmacy, loneliness and ADL/IADL appeared relevant and were retained in the final model. The association between number of conditions and QoL in the base model was -2.44 [95% CI: -2.72; -2.16], while this association was -0.76 [95%CI: -0.97; -0.54] after all relevant factors were included.

**Conclusion:**

Factors beyond the socio-economic circumstances play an important role in explaining the association between number of conditions and QoL. These factors should be considered to better estimate the impact of chronic diseases on QoL, and for improving patient care.

## Introduction

Ageing society has become a growing phenomenon worldwide [1]. One of the most frequent companions of an increasing age is an accumulation of diseases, and living with multiple conditions in advanced age has become the norm [2]. Multimorbidity is usually defined as coexistence of two or more chronic conditions [3]. It is associated with increased disability and functional decline, and increased health care costs [4]. The negative association between multimorbidity and quality of life (QoL) has also been well documented in the past decades [4, 5]. Quality of life is a good indicator of patient satisfaction with quality and availability of care [6], as well as their capability to lead a fulfilling life despite impaired health. This may be particularly challenging for people with multiple health conditions.

The relationship between multimorbidity and QoL is most often explored accounting for demographic and socio-economic factors, while other elements which may also shape this association and obscure the true effect of multimorbidity, are rarely accounted for [5]. Here, we investigated the role of some of these factors, in addition to socio-economic indicators with the aim to estimate their relevance in this association and foster their consideration when drafting a personalised care plan for a patient.

For instance, presence of symptoms such as pain, fatigue, dizziness or falling, which often accompany medical conditions, deteriorates QoL [7]. In the absence of a widely agreed multimorbidity definition and which conditions should it contain [8], symptoms often make part of it [9, 10]. Covered under umbrella of multimorbidity, their sole impact on QoL is hard to assess.

Treatment burden is a relatively new and unexplored concept [11] related to the notion of minimally disruptive medicine which aims to adjust treatment to the capacities of a patient [12]. The emerging literature provides a clear understanding that frequent visits to different health care providers, conflicting recommendations and multiple medication use, among others, give additional weight to daily functioning in the context of multimorbidity [13–16]. Still, treatment burden is not accounted for when exploring the association between multimorbidity and QoL [5].

Further, it has been first pointed out by Fortin et al. in 2006 [17], that perceived social support plays a key role in improving QoL in patients with multiple conditions. Since then, exploring the role of social support on QoL in the context of multimorbidity has gained in interest [18]. However, with some exceptions [10], taking social support into account when associating multimorbidity with QoL has not become a common practice.

Similarly, difficulties with activities of daily living (ADL) and instrumental activities of daily living (IADL) carry a loss of independence, leading to lower self-esteem, possibly isolation, and ultimately deterioration of life quality [19]. Yet, not many studies control for these when exploring this relationship [20].

**Fig 1:**
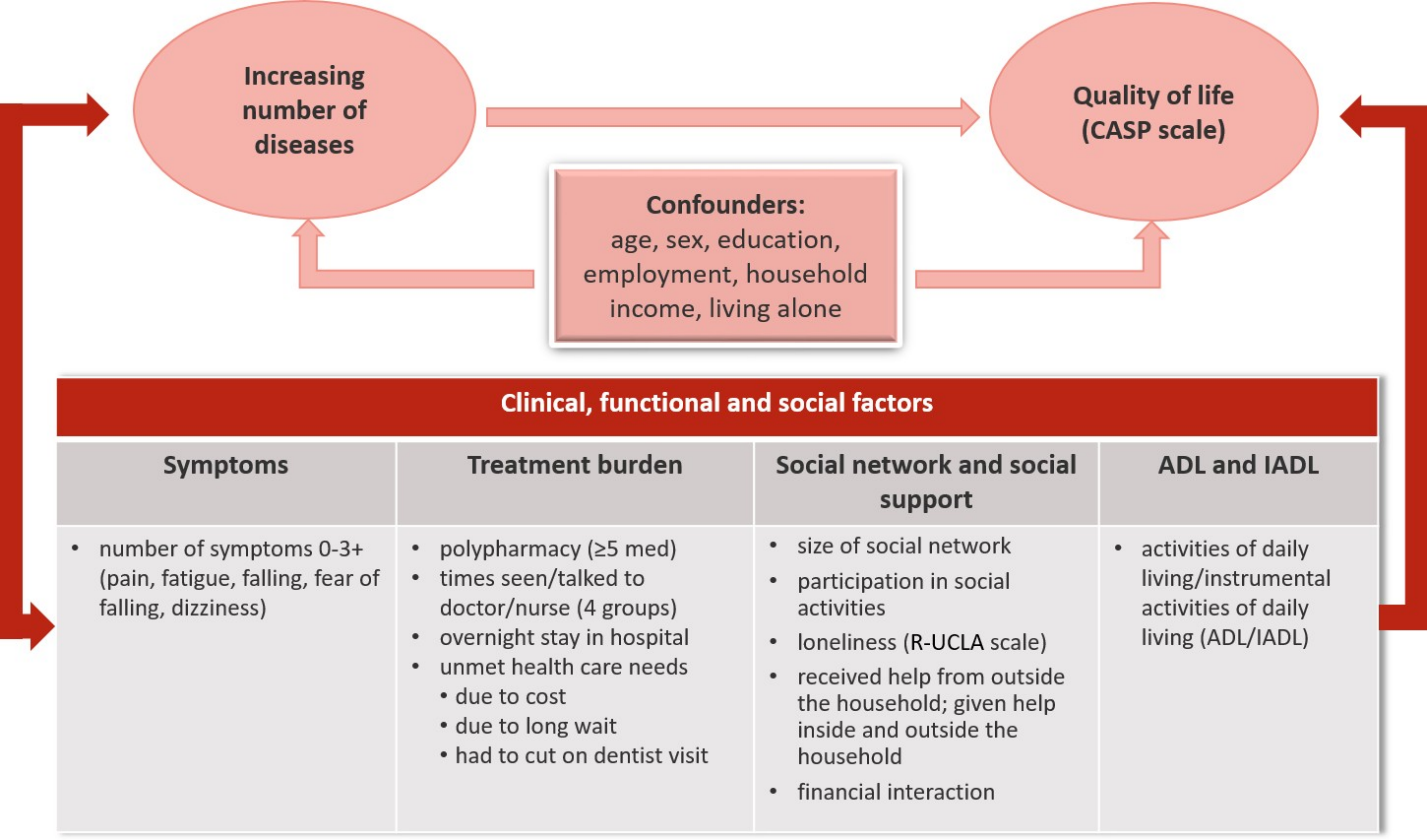
Conceptual framework describing clinical, functional and social factors to test for relevance in the association between number of diseases and quality of life adjusted for confounders.

While negative impact of most of the mentioned factors on QoL is familiar, their role in the relationship between multimorbidity and QoL is insufficiently explored. This study aims to fill this gap by introducing the factors separately in the analysis of association between number of diseases and QoL, in a large study sample comprising several populations of older adults across Europe. For practical reasons, these factors are later referred to as clinical (symptoms and indicators of treatment burden), functional (ADL/IADL) and social factors (social network and social support), or factors of interest. The conceptual framework of the study is presented in Fig 1.

## Materials and methods

### Study population

We performed a cross-sectional analysis on wave 6 of the population-based Survey of Health, Ageing and Retirement in Europe (SHARE) [21]. SHARE is a panel survey containing information on socio-economic status, health, family circumstances and social network, across several European countries and Israel [22]. Eighteen countries participated in the wave 6 face-to-face interview in 2015 [21]. Eligible participants were age 50 and older residing in participating countries at the time of the interview. This also included individuals living in nursing homes/institutions; their proportion however varied significantly, depending on the country’s sampling frame coverage [23].

### Study population characteristics

#### Morbidity assessment

The presence of 17 specified conditions or groups of conditions, was self-declared and determined with the question: “Has a doctor ever told you that you had/ Do you currently have any of the conditions on this card? With this we mean that a doctor has told you that you have this condition, and that you are either currently being treated for or bothered by this condition” [24]. List of conditions is presented in the Table 1.

**Table 1:** List of conditions.

• heart attack including myocardial infarction or coronary thrombosis or any other heart problem including congestive heart failure; • high blood pressure or hypertension; • high blood cholesterol; • stroke or cerebral vascular disease; • diabetes or high blood sugar; • chronic lung disease such as chronic bronchitis or emphysema; • cancer or malignant tumour, including leukaemia or lymphoma, but excluding minor skin cancers; • stomach or duodenal ulcer, peptic ulcer; • Parkinson disease;	• cataracts; • hip fracture; • other fractures; • Alzheimer's disease, dementia, organic brain syndrome, senility or any other serious memory impairment; • other affective or emotional disorders, including anxiety, nervous or psychiatric problems; • rheumatoid arthritis; • osteoarthritis, or other rheumatism; • chronic kidney disease; • other conditions, not yet mentioned*

*Other conditions, not mentioned on the list could not be specified or retrieved due to SHARE data-protection policy. Therefore, any declared additional disease(s) were counted as one, and a total number of 18 conditions was considered.

#### Quality of life assessment

Quality of life was assessed by the Control, Autonomy, Self-Realization and Pleasure (CASP) scale [25], in its shorter 12 question version, CASP-12v1 [26]. The questionnaire intends “to cover the active and beneficial experiences of later life rather than simply focus on the medical and social care issues” [26]. The CASP score ranges from 12 to 48, a lower number indicating worse QoL [26]. We rescaled CASP to range 0–100 to enable easier comparability with studies using other QoL scales. A change of 1 on the original CASP corresponds to a change of 2.78 on rescaled CASP.

#### Socio-economic characteristics

The answers on the highest school certificate obtained [24] were standardized by 1997 International Standard Classification of Education (ISCED97) and categorised in three levels: low, medium, high [27]. To describe current employment situation, participants could choose between employed or self-employed, retired, unemployed, permanently sick or disabled, homemaker or other [24]. The household income variable (used in quantiles) was provided by SHARE Central team [28]. The living situation was obtained from the household size. Household size 1 indicated living alone; >1 implied not living alone.

#### Factors of interest

*Symptoms*. Participants reported if being bothered by any of the following five symptoms in the past minimum six months: pain, dizziness, faints or blackouts, falling, fear of falling and fatigue [24]. Symptoms were summed to range 0 to 3+.

*Treatment burden indicators*. The SHARE study did not include a treatment burden questionnaire. Therefore, we looked for variables that could serve as indicators of the burden of treatment, relying on the Multimorbidity Treatment Burden Questionnaire [16] and other relevant literature [13, 14]. Identified indicators were: polypharmacy, unmet health care needs and utilisation of health care. To assess polypharmacy, participants reported taking or not at least five drugs per day, including prescribed drugs, drugs bought without prescription or dietary supplements [24]. Cost and waiting time to receive medical care were operationalised as unmet needs. They were assessed by asking whether in the last 12 months interviewees had to refrain from seeing a doctor or dentist due to cost or long waiting time [24]. Health care utilisation was measured with two approaches: number of times in the past year a participant talked to/saw a doctor or qualified nurse, what included emergency admissions and outpatient clinic visits, but excluded visits to a dentist and hospital stays (grouped as: 0, 1 or 2, 3–5, 6–11 and ≥12 times); and a binary report regarding overnight stay(s) in the hospital in the past year [24].

*Social network and social support*. The size of participants’ network and social participation were used to describe characteristics of the network [29]. The size of the social network was assessed by asking with whom participants most often discussed important things in the past year [24]. The size ranged from zero to seven, while we used 4+ as the upper limit. Participants were considered socially active if they were involved in at least one of the following activities in the past 12 months: voluntary or charity work; educational or training course; sport, social or other kind of club; or taken part in a political or community-related organisation [24].

Positive social interaction is also considered as one of the categories of social support, together with provision and receipt of personal and financial help [29, 30]. Participants could report whether they have received help from or have given help to a family member outside the household, a friend or neighbour, in the past year. The help included assistance with ADL and IADL [24]. Providing help inside the household was evaluated by asking about help with personal care a participant provided on a regular basis in the last 12 months inside the household [24]. To assess financial interaction, participants reported whether given or received a financial or material gift in amount of 250 euros or more in the previous year to/from any person inside or outside the household [24].

Loneliness served as an indicator for perceived social support [31]. It was assessed by the R-UCLA 3-items scale [32]. The score ranged from three to nine; nine implying very lonely.

*Activities and instrumental activities of daily living*. To assess problems with ADL and IADL, participants could declare whether they have difficulties with basic daily (n = 6) or more complex tasks (n = 9) and which they expect to last more than three months [24].

Activities and instrumental activities of daily living were combined into a single binary variable where participants were considered having problems if they were limited with at least one of the activities [33–35].

### Statistical analyses

We used mean, standard deviation and percentages to summarize the data. To estimate the relationship between number of diseases and QoL, a multilevel mixed-effects linear regression was performed. Study design of SHARE required a 3-level model (individual, household and country). Random coefficient model was compared to the random intercept model. Likelihood ratio test showed p-value<0.0001, therefore the random coefficient model was chosen, which allows both the intercepts and the slopes between number of diseases and QoL to vary at the country level. All analyses were performed in Stata 14.0.

The base model was adjusted for sex, age, education, employment, household income and living alone [36]. Fractional polynomials (FP) on age and number of diseases were used to test for possible nonlinear relationships with the outcome on the base model [37]. Given that QoL increases until approximately age of 67, followed by a decline, the second degree FP model for age was selected with 2 powers, of 0.5 and 2. In contrast, the linear model was selected for the number of diseases. Linearity was also confirmed by comparing coefficients of number of chronic conditions between disease groups when number of diseases was treated as a categorical variable [38].

Clinical, functional and social factors were tested one at the time in the base model. All factors, which inferred a change of 15% or more of the coefficient of the number of diseases compared to the coefficient in the base model, were retained in the final model.

To assess potential difference in the association in men and women, we tested the interaction between sex and number of conditions in the final model.

Individual countries’ intercepts and slopes were examined to gain insight into possible difference.

To test a potential overlap between independent variables, t-test and ANOVA were applied to evaluate associations between number of conditions and all other covariates, as well as among all factors of interest included in the final model. Collinearity was tested in the final model with Variance Inflation Factor (VIF).

In addition, a sensitivity analysis was performed by re-running models with each factor of interest separately, removing the category “other diseases” from the disease count, counting the 17 specified conditions.

## Results

The study population included a total of 67 179 individuals, with mean age 68.0 (SD±10). The majority were females (56.0%), and 21.7% lived alone. Most participants had low educational level (41.2%) and were retired (59.4%). The number of chronic conditions ranged from 0 to 13. Almost half (49.6%) of participants lived with multimorbidity, whereas 28.0% of participants had only one condition and 22.4% had none. People with multimorbidity were older, had lower education and lower household income compared to people with no multimorbidity; they also reported having mostly two or three conditions (Table 2).

**Table 2:** Socio-economic characteristics of the study sample.

Variable and variable categories	Total population	Without multimorbidity	With multimorbidity
		(<2 chronic conditions)	(≥2 chronic conditions)
	N (%)*	N (%)*	N (%)*
Total number of participants (age 50+)	67 179 (100.00)	33 795 (50.42)	33 235 (49.58)
Sex			
Male	29 576 (44.03)	15 577 (46.09)	13 934 (41.93)
Female	37 603 (55.97)	18 218 (53.91)	19 301 (58.07)
Age (mean, SD)	67.95 (±10)	65.28 (±9.4)	70.66 (±9.8)
Age groups			
50–54	6 307 (9.39)	4 577 (13.55)	1 716 (5.16)
55–59	10 327 (15.37)	6 768 (20.03)	3 537 (10.64)
60–64	11 985 (17.84)	7 032 (20.81)	4 930 (14.83)
65–69	11 910 (17.73)	5 855 (17.33)	6 039 (18.17)
70–74	9 535 (14.19)	3 951 (11.69)	5 566 (16.75)
75–79	7 792 (11.60)	2 720 (8.05)	5 058 (15.22)
80+	9 317 (13.87)	2 888 (8.55)	6 387 (19.22)
Living alone			
Yes	14 568 (21.69)	6 214 (18.39)	8 304 (24.99)
No	52 611 (78.31)	27 581 (81.61)	24 931 (75.01)
Educational level			
High	14 459 (21.85)	8 708 (26.14)	5 727 (17.51)
Medium	24 462 (36.97)	13 074 (39.25)	11 339 (34.66)
Low	27 240 (41.17)	11 531 (34.61)	15 647 (47.83)
Employment status			
Employed or self-employed	16 001 (24.16)	11 638 (34.73)	4 357 (13.32)
Retired	39 349 (59.40)	16 873 (50.35)	22 460 (68.68)
Homemaker	5 781 (8.73)	2 743 (8.19)	3 034 (9.28)
Unemployed	1 828 (2.76)	1 107 (3.30)	721 (2.20)
Permanently sick or disabled	2 027 (3.06)	575 (1.72)	1 452 (4.44)
Other	1 255 (1.89)	573 (1.71)	680 (2.08)
Household income (quintile)			
5 (highest)	13 294 (19.79)	8 273 (24.48)	4 992 (15.02)
4	13 376 (19.91)	7 180 (21.25)	6 165 (18.55)
3	13 477 (20.06)	6 477 (19.17)	6 970 (20.97)
2	13 504 (20.10)	5 880 (17.40)	7 596 (22.86)
1 (lowest)	13 528 (20.14)	5 985 (17.71)	7 512 (22.60)
Chronic disease groups			
0	15 030 (22.42)	15 030 (44.47)	na
1	18 765 (27.99)	18 765 (55.53)	na
2	14 236 (21.24)	na	14 236 (42.83)
3	9 311 (13.89)	na	9 311 (28.02)
4	5 101 (7.61)	na	5 101 (15.35)
5	2 531 (3.78)	na	2 531 (7.62)
6	1 193 (1.78)	na	1 193 (3.59)
7	509 (0.76)	na	509 (1.53)
8	200 (0.30)	na	200 (0.60)
9	99 (0.15)	na	99 (0.30)
10	33 (0.05)	na	33 (0.10)
11	14 (0.02)	na	14 (0.04)
12	7 (0.01)	na	7 (0.02)
13	1 (0.00)	na	1 (0.00)

*percentages do not include missing values.

Four out of 10 people reported having no symptoms and almost eight in 10 used <5 drugs per day. Participants mostly declared not having unmet health care needs, 28.7% contacted doctor/nurse 3–5 times in the past year and 15.4% had at least one overnight hospital stay. They mainly had two confidents (25.9%) and 55.9% did not feel lonely. The majority did not receive nor gave help outside the household (77.5% and 73.2%), while 6.5% provided help inside the household. Financial interaction occurred among 31.6% of participants. Over half of the SHARE population was not socially active (60.3%), while 22.0% had at least one difficulty in daily life (ADL or IADL). Compared with people with no multimorbidity, participants with two or more diseases had more symptoms, reported much more often taking ≥ 5 medications daily, declared more having unmet health care needs due to cost or waiting time and seeked medical assistance more regularly with more frequent overnight hospital stays. The network size between the two groups was comparable, but people with multimorbidity felt lonelier, participated bit less in social activities and received more help from others. They reported having more difficulties with ADL/IADL (Table 3).

**Table 3:** Clinical, functional and social factors of the study sample.

Variable and variable categories	Total population	Without multimorbidity	With multimorbidity
		(<2 chronic conditions)	(≥2 chronic conditions)
	N (%)*	N (%)*	N (%)*
Number of symptoms			
0	27 505 (41.05)	19 582 (57.99)	7 914 (23.83)
1	20 812 (31.06)	9 955 (29.48)	10 846 (32.66)
2	10 865 (16.22)	3 210 (9.51)	7 651 (23.04)
3+	7 821 (11.67)	1 019 (3.02)	6 798 (20.47)
Polypharmacy			
0–4	52 062 (77.70)	31 798 (94.15)	20 245 (60.97)
≥5	14 943 (22.30)	1 975 (5.85)	12 959 (39.03)
Unmet needs in the last 12 months			
Due to cost	3 164 (4.72)	1 068 (3.16)	2 095 (6.31)
yes			
no	63 809 (95.28)	32 689 (96.84)	31 091 (93.69)
Due to long waiting time	6 351 (9.48)	2 239 (6.63)	4 111 (12.39)
yes			
no	60 626 (90.52)	31 516 (93.37)	29 081 (87.61)
Postponed dentist visit	5 432 (8.11)	2 253 (6.67)	3 177 (9.57)
yes			
no	61 566 (91.89)	31 514 (93.33)	30 024 (90.43)
Times seen or talked to a doctor or qualified nurse in the last 12 months			
0	7 051 (10.62)	5 733 (17.08)	1 316 (4.01)
1 or 2	15 098 (22.74)	10 703 (31.89)	4 390 (13.38)
3 to 5	19 043 (28.69)	9 595 (28.59)	9 441 (28.78)
6 to 11	13 529 (20.38)	4 728 (14.09)	8 795 (26.81)
≥12	11 665 (17.57)	2 801 (8.35)	8 862 (27.01)
Hospital overnight stay in the last 12 months			
yes	10 299 (15.37)	3 085 (9.13)	7 210 (21.71)
no	56 708 (84.63)	30 688 (90.87)	25 995 (78.29)
Social network size			
0	1 614 (2.70)	793 (2.63)	796 (2.69)
1	15 078 (25.19)	7 976 (26.44)	7 071 (23.88)
2	15 506 (25.91)	7 724 (25.60)	7 770 (26.24)
3	12 874 (21.51)	6 289 (20.85)	6 579 (22.22)
4+	14 780 (24.69)	7 384 (24.48)	7 394 (24.97)
Loneliness (R-UCLA scale 3–9)			
not lonely	35 771 (55.88)	20 395 (62.24)	15 367 (49.21)
4	12 084 (18.88)	6 053 (18.47)	6 027 (19.30)
5	6 805 (10.63)	2 944 (8.98)	3 860 (12.36)
6	5 116 (7.99)	2 072 (6.32)	3 042 (9.74)
7	2 066 (3.23)	703 (2.15)	1 361 (4.36)
8	980 (1.53)	318 (0.97)	662 (2.12)
very lonely	1 193 (1.86)	282 (0.86)	909 (2.91)
Received help with ADL/IADL from someone outside the household in the last 12 months			
yes	15 074 (22.49)	5 470 (16.20)	9 591 (28.89)
no	51 956 (77.51)	28 293 (83.80)	23 603 (71.11)
Given help with ADL/IADL to someone outside the household in the last 12 months			
yes	17 954 (26.80)	10 144 (30.05)	7 808 (23.52)
no	49 029 (73.20)	23 613 (69.95)	25 384 (76.48)
Given help with personal care to someone inside the household in the last 12 months			
yes	4 348 (6.50)	1 772 (5.26)	2 574 (7.77)
no	62 514 (93.50)	31 913 (94.74)	30 537 (92.23)
Financial interaction in the last 12 months			
yes	20 951 (31.62)	11 015 (33.07)	9 909 (30.14)
no	45 300 (68.38)	22 291 (66.93)	22 963 (69.86)
Participation in social activities in the last 12 months			
yes	25 319 (39.73)	14 528 (44.49)	10 783 (34.73)
no	38 406 (60.27)	18 129 (55.51)	20 268 (65.27)
Having difficulties with ADL or IADL (and expected to last ≥3 months)			
yes	14 748 (22.00)	3 398 (10.06)	11 337 (34.12)
no	52 286 (78.00)	30 386 (89.94)	21 885 (65.88)

*percentages do not include missing values.

ADL = Activities of Daily Living.

IADL = Instrumental Activities of Daily Living.

After all factors of interest were introduced in the base model separately, symptoms, polypharmacy, loneliness and ADL/IADL appeared significant; based on minimum of 15% coefficient estimate change (Table 4).

**Table 4:** Association between number of conditions and quality of life: Estimates from multilevel models adjusted for potential confounders^a^.

Models	β [95%CI] for number of chronic conditions
Base model (adjusted for age, sex, education, employment, household income and living alone)	-2.44 [-2.72; -2.16]
Base model + symptoms*	-1.30 [-1.56; -1.03]
Base model + polypharmacy*	-2.00 [-2.29; -1.71]
Base model + unmet need (cost)	-2.35 [-2.60; -2.09]
Base model + unmet need (long wait)	-2.34 [-2.61; -2.08]
Base model + unmet need (cut on dentist visits)	-2.36 [-2.63; -2.09]
Base model + times talked to doctor	-2.08 [-2.36; -1.80]
Base model + overnight stay	-2.33 [-2.62; -2.05]
Base model + social network size	-2.49 [-2.77; -2.22]
Base model + loneliness (R-UCLA scale)*	-1.91 [-2.12; -1.71]
Base model + received care outside HH	-2.35 [-2.63; -2.07]
Base model + given care outside HH	-2.43 [-2.71; -2.15]
Base model + given care inside HH	-2.43 [-2.72; -2.15]
Base model + financial interaction	-2.44 [-2.72; -2.16]
Base model + social activities	-2.40 [-2.68; -2.11]
Base model + ADL/IADL*	-1.88 [-2.16; -1.60]

CI = Confidence Interval.

HH = Household.

ADL = Activities of Daily Living.

IADL = Instrumental Activities of Daily Living.

^a^ Each factor of interest was separately added in the base model.

*Significant change (number of conditions coefficient changed for ≥15% compared to the coefficient in the base model).

The base model, adjusted for socio-economic factors only, presented negative association between number of conditions and QoL of -2.44 [95% CI: -2.72; -2.16] (Table 4). Above mentioned significant factors of interest were all added to the base model to form the final model. The final model presented now the association of -0.76 [95%CI: -0.97; -0.54] (Table 5). After factors were added, the negative association between chronic conditions and QoL was reduced by 69% compared to the base model, with symptoms contributing the most to this reduction. Namely, adding symptoms in the base model reduced the coefficient of number of chronic conditions by 46.9% (from -2.44 [95% CI: -2.72; -2.16] to -1.30 [95%CI: -1.56; -1.03]). Adding only ADL/IADL difficulties in the base model, lessened the strength of the negative association by 22.8% (to -1.88 [95%CI: -2.16; -1.60]) while adding loneliness weakened the strength by 21.5% (to -1.91 [95%CI: -2.12; -1.71]). Including only polypharmacy reduced the strength of the association by 18% compared to the base model (to -2.00 [-2.29; -1.71]). Adding variable “times talked to doctor”, the coefficient of number of chronic conditions changed by 14.6% (to -2.08 [95%CI: -2.36; -1.80]). While this change did not cross our set threshold of 15% for relevance, this finding insinuated a large influence that number of medical visits may have on QoL in patients with multimorbidity (Table 4).

**Table 5:** Final multivariable model for quality of life.

Variable	β [95% CI]	Standardised β [95% CI]
Number of chronic conditions	-0.76 [-0.97;-0.54]	-1.23 [-1.57;-0.89]
Sex (female vs. male)	0.84 [0.65;1.03]	0.84 [0.65;1.03]
Age^0.5^	8.33 [6.63;10.03]	5.02 [3.99;6.05]
Age^2^	-0.004 [-0.005;-0.003]	-5.32 [-6.33;-4.32]
Education (ref. high)		
medium	-0.65 [-0.93;-0.38]	-0.65 [-0.93;-0.38]
low	-2.15 [-2.45;-1.84]	-2.15 [-2.45;-1.84]
Employment (ref. employed)		
retired	0.17 [-0.17;0.51]	0.17 [-0.17;0.51]
homemaker	-0.51 [-0.95;-0.07]	-0.51 [-0.95;-0.06]
other	-1.55 [-2.33;-0.77]	-1.55 [-2.33;-0.77]
unemployed	-2.46 [-3.08;-1.84]	-2.46 [-3.08;-1.83]
permanently sick or disabled	-3.20 [-3.85;-2.55]	-3.20 [-3.85;-2.55]
Living alone (yes vs. no)	2.62 [2.32;2.92]	2.62 [2.32;2.92]
Income in quintiles (ref. highest)		
4	-1.74 [-2.10;-1.38]	-1.74 [-2.10;-1.38]
3	-2.82 [-3.19;-2.45]	-2.82 [-3.19;-2.45]
2	-3.87 [-4.25;-3.48]	-3.87 [-4.25;-3.48]
1 (lowest)	-5.79 [-6.19;-5.39]	-5.79 [-6.19;-5.39]
Symptoms (ref. 0)		
1	-3.04 [-3.28;-2.80]	-3.04 [-3.28;-2.80]
2	-5.99 [-6.32;-5.67]	-6.00 [-6.32;-5.67]
3+	-8.73 [-9.15;-8.31]	-8.73 [-9.15;-8.31]
Polypharmacy (≥5 drugs vs. <5 drugs)	-1.43 [-1.72;-1.14]	-1.43 [-1.72;-1.14]
Loneliness (scale 3–9) (ref. not lonely)		
4	-4.36 [-4.63;-4.10]	-4.36 [-4.63;-4.10]
5	-8.91 [-9.25;-8.57]	-8.91 [-9.25;-8.57]
6	-12.99 [-13.38;-12.60]	-12.99 [-13.38;-12.60]
7	-16.22 [-16.81;-15.63]	-16.22 [-16.81;-15.63]
8	-18.31 [-19.14;-17.47]	-18.31 [-19.14;-17.47]
9 (very lonely)	-23.49 [-24.27;-22.72]	-23.50 [-24.27;-22.72]
Difficulties with ADL/IADL (yes vs. no)	-4.27 [-4.56;-3.97]	-4.27 [-4.56;-3.97]
Constant	31.71 [20.90;42.52]	80.88 [78.47;83.28]

CI = Confidence Interval.

ADL = Activities of Daily Living.

IADL = Instrumental Activities of Daily Living.

Age^0.5^ = Fractional Polynomial 1.

Age^2^ = Fractional Polynomial 2.

Our final multivariable model (Table 5) showed that increasing number of symptoms, polypharmacy or having at least one difficulty in daily living was negatively associated with QoL. The gradient of loneliness disclosed a stronger negative association with QoL, with -23.49 [95%CI: -24.27;-22.72] QoL score for very lonely participants compared to those not lonely.

No strong associations were detected between tested variables. There was no collinearity in the final model (highest VIF = 1.30).

Variables had in general a low number of missing values (mostly <0.5%; education, employment, number of times talked to doctor/nurse and financial interaction had each <2%). The highest number of missing values was for loneliness (4.7%), participation in social activities (5.1%), CASP (6.7%) and social network size (10.9%). No imputation was done for the original analyses. To verify our findings, additional analyses were performed by recoding missing values to a category for all categorical variables with >1% of missings; findings were robust across the models.

The interaction term between sex and number of conditions in the final model showed p-value of 0.029. Stratified analyses by sex for the final model showed stronger negative association between number of conditions and QoL for men (-0.83 [95%CI: -1.08; -0.57]) compared to women (-0.77 [95%CI: -1.04; -0.49]) (S1 Table).

**Fig 2:**
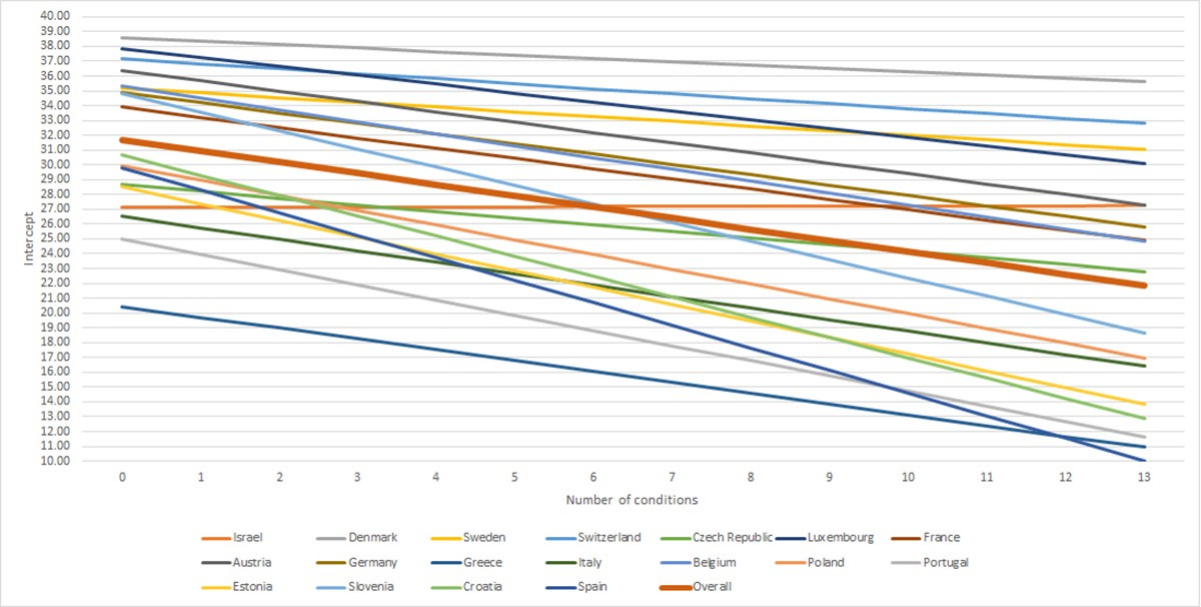
Countries’ intercepts and slopes.

Random effects in the final multivariable model showed significant variations of the mean QoLs across both countries and households, while more variations were observed across households. Association between number of diseases and QoL was also significantly different across countries. As presented in Fig 2 and S2 Table, Denmark, Luxembourg and Switzerland had the highest QoL. Spain, Croatia and Slovenia presented the strongest negative association between number of conditions and QoL.

Sensitivity analysis performed by removing the category “other diseases” from the disease count amplified the significance of number of contacts with a doctor or qualified nurse in the association between number of diseases and QoL, reaching a predefined level of relevant coefficient estimate change of 15% (15.9%) (S3 Table).

## Discussion

Our study shows that clinical, functional and social factors significantly weaken the association between multimorbidity and QoL, after adjustment for commonly considered socio-economic factors [5]. Specifically, number of symptoms, limitations with ADL/IADL, perceived social support and polypharmacy accounted for a large portion of the association between number of diseases and QoL.

In the literature, symptoms, like pain, fatigue or dizziness are frequently counted in multimorbidity records [9, 10], due to current inconsistency in multimorbidity definition on whether only medical diagnosis or also conditions and symptoms should be included [8]. This not only hampers comparability between studies, but also prevents an estimation of the true impact of symptoms on QoL. Increasing number of symptoms strongly contributed to deterioration of QoL in our multivariable model. This could reflect the severity of diseases or undiagnosed conditions. The finding emphasizes a potential significance in managing symptoms for maintaining satisfying QoL, what can be addressed during the decision-making process in a patient centered care approach [8]. We acknowledge that there is a certain overlap with the symptoms included in the study with those belonging to geriatric syndrome, such as falls [39]. However, considering mean age of our population and that falls increasingly appear after age of 70 [40], we did not make this distinction in our assessment.

The impact of ADL and IADL on QoL in multimorbidity is documented earlier [20]. While some seem to suggest a mediating role of these factors in the relationship between chronic diseases and QoL [41], others argue that difficulties with daily activities rather interact with chronic conditions, highlighting the less strong association between number of diseases and QoL for individuals with ADL difficulties [20]. These, as well as our findings warrant more scientific evidence. This might be particularly important for planning a comprehensive care for patients with multimorbidity, as focusing only on diseases may not necessarily bring desired benefit to QoL if capability to manage daily activities is not improved [20].

Loneliness as an indicator of perceived social support altered the relationship between number of diseases and QoL. This supports findings by Fortin et al. [17] in a primary care setting and reemphasises the need for controlling for perceived social support when exploring this question. We used the loneliness scale for this purpose; however, scales which measure perceived social support directly could be even better suited. Interventions to prevent loneliness among elderly population focus largely on promoting group activities where participants socialise while taking part in the activity of choice, or they encourage closer individual interactions in the groups of two [42]. These examples could induce more action at the regional and national levels to tackle loneliness.

Polypharmacy is one of the main indicators of treatment burden [15]. The burden is related to the inconvenience in administering multiple medications, their side effects and adverse events, and stigma [15]. Apart from polypharmacy, none of the other tested elements of treatment burden met the threshold for relevance, although number of contacts with a doctor notably interfered in the association in the original findings and it was significant in the sensitivity analysis. Having to comply with multiple visits to healthcare providers requires scheduling and attending appointments, spread often over several occasions, arranging transportation, waiting on treatment, etc. [11, 14], what certainly may impose additional burden on a patient. The National Institute for Care and Excellence’s guide for clinical assessment and management of multimorbidity [43] points out a number of measures which serve to identify and alleviate burden of treatment in patients with multimorbidity. We were unfortunately not able to assess treatment burden as a whole, as treatment burden questionnaire was not employed in the SHARE study. We however intended to raise awareness that additional burden, derived from the disease management interferes significantly in the relationship between multimorbidity and QoL, and point out the relevance of accounting for treatment burden when next exploring this question.

A comparable study on the association between multimorbidity and QoL using SHARE-data which adjusted on socio-economic factors only [36], demonstrated a slightly stronger negative association than the one presented in our base model (our non-rescaled CASP coef. for base model -0.88 [95%CI: -0.98; -0.78]). One of the reasons might be that this study used the EURO-D depression scale to assess psychological status of participants. Adjusting for additional factors in our study explained the further weakening of the association.

We compared also the strength of the association in our base model to the slopes observed in the meta-analysis studies that applied other QoL scales. Our association was less strong compared to EQ-5D and SF-6D and physical domains of SF-non-preference based scales and WHOQoL-BREF scale, but stronger compared to psychological domains of the latter two scales [5]. These differences may have derived from difference between the scales, adjustment factors and study designs. Our final fully adjusted model, however, showed significantly less strong association compared to all scales. We argue that this is largely due to controlling for various other relevant factors in our final model, what resulted in weaker association between number of diseases and QoL.

This study was performed on a large sample of adults comprising several European countries. Thanks to comprehensiveness of the data, this is a rare study to test for various potential confounders in the relationship between multimorbidity and QoL, based on a substantial number of different covariates. Moreover, one of its main contributions could be consideration of indicators of treatment burden in this association [5].

Some limitations nevertheless, entail discussion. Medical conditions were self-reported and although self-report could provide a solid estimate of diseases burden, SHARE participants may have understated or overstated their morbidity status. Further, the list of conditions was limited, and while there was an option of adding unspecified conditions, those could not be retrieved. We counted the category “other” as an additional disease as we wanted to acknowledge that participant found necessary to declare an additional concern; however, it is uncertain whether conditions reported there were actually stated previously from the list, how many were reported, or whether those were disabilities or symptoms. A sensitivity analysis excluding the “other” diseases was performed to strengthen our findings. Also some diseases were grouped, preventing more precise reporting of a number of diagnosis. Nonetheless, the list included the most relevant and prevalent conditions. Further, even though the CASP scale seems to be increasingly used, it remains yet one of the rarer applied instruments. This prevents wider comparability. However, in the absence of a multimorbidity-specific QoL questionnaire, this scale intended for elderly, may have well been most appropriate choice when it comes to grasping relevant dimensions of QoL. The severity of diseases was not assessed in our study; this could have provided a more comprehensive understanding of a disease burden. Lastly, the cross-sectional design of the study does not allow to infer causality, and reverse causation (e.g. loneliness) cannot be excluded.

Several factors did not present significant confounding role in the association between multimorbidity and quality of life in our study. The reasons may be numerous. For instance, cost or waiting time were in general not reported very often as obstacles to access care. This may be due to Europe’s generally universal health care coverage that certainly alleviates some of the patients’ strains in this regard. The role of these factors may be amplified in health care systems with a different organisation. Also, to operationalise treatment burden we used the number of contacts with health professionals and number of overnight hospital stays as an indicator. These may have not been the ideal measures to assess this; however the number of contacts in the sensitivity analysis showed significance.

Likewise, not all indicators of social support confounded the association between number of diseases and QoL. It is possible that the size of social network or participation in social activities did not mirror the intimacy of the social interaction, what was instead better captured with loneliness. Similar may be the case for the personal care and financial interaction.

We have demonstrated that controlling only for socio-economic factors is not sufficient for quantifying the relationship between number of diseases and QoL and that including other covariates is warranted for a more precise estimation. As QoL is set as one of the core outcomes for multimorbidity research [44], some of the factors discussed in this manuscript may serve as suggestion for future investigation. Exploring their role as mediators in this context could potentially be of interest; that information may assist in planning targeted preventive measures. As we were only able to rely on self-reported conditions, comparative studies of a longitudinal design with verified diagnoses through e.g. medication use, could provide additional clarification. And importantly, accounting for severity of diseases and time living with conditions would add to the body of knowledge.

Countries displayed substantial variations in the strength of the association between number of diseases and QoL which could reflect economic, cultural or health care system differences. Future research is warranted to explore these findings with the aim of magnifying the best practices and sharing the knowledge.

Lastly, it was our aim to evaluate how factors we found relevant and available in the SHARE database intervene in the association between multimorbidity and QoL as a whole; however, the final model showed difference between men and women. It is worthwhile taking closer look on what may be causing this discrepancy.

## Conclusions

Our findings demonstrate that besides morbidity and socio-economic characteristics, other factors such as clinical, functional and social factors explain deterioration of QoL. These elements should be accounted for when studying the relationship between multimorbidity and QoL. Better estimating the impact of multiple conditions, as well as other related factors on the overall wellbeing and QoL of patients with multimorbidity will consequently enable more holistic approach when planning the care.

## Supporting information

S1 Table Analyses stratified by sex for the final model. (XLSX)

S2 Table Countries’ intercepts and slopes. (DOCX)

S3 Table Sensitivity analysis without category “other diseases” in the disease count. (XLSX)
