# Supplementary material for: Role of clinical, functional and social factors in the association between multimorbidity and quality of life: Findings from the Survey of Health, Ageing and Retirement in Europe (SHARE)
Source: PLoS One. 2020 Oct 20;15(10):e0240024. doi: 10.1371/journal.pone.0240024 (PMC7575102; doi:10.1371/journal.pone.0240024)
Supplement: S2 Table — (DOCX) [file pone.0240024.s002.docx]

S2 Table: Countries’ intercepts and slopes

| **Country** | **intercept** | **slope** |
| --- | --- | --- |
| Israel | 27.12 | 0.01 |
| Denmark | 38.56 | -0.23 |
| Sweden | 35.18 | -0.32 |
| Switzerland | 37.16 | -0.34 |
| Czech Republic | 28.66 | -0.45 |
| Luxembourg | 37.84 | -0.60 |
| France | 33.89 | -0.69 |
| Austria | 36.37 | -0.70 |
| Germany | 34.92 | -0.70 |
| Greece | 20.44 | -0.73 |
| Italy | 26.53 | -0.78 |
| Belgium | 35.30 | -0.80 |
| Poland | 29.95 | -1.00 |
| Portugal | 24.99 | -1.03 |
| Estonia | 28.52 | -1.13 |
| Slovenia | 34.82 | -1.25 |
| Croatia | 30.65 | -1.37 |
| Spain | 29.83 | -1.52 |
| *Overall* | *31.71* | *-0.76* |
